# Supplementary material for: Reconciling Mining with the Conservation of Cave Biodiversity: A Quantitative Baseline to Help Establish Conservation Priorities
Source: PLoS One. 2016 Dec 20;11(12):e0168348. doi: 10.1371/journal.pone.0168348 (PMC5173368; doi:10.1371/journal.pone.0168348)
Supplement: S1 Dataset — (ZIP) [file pone.0168348.s002.zip › Taxa/Serra Norte/SN_2013/N4E/Lista N4E-05.pdf]

CAVIDADE N4E-0005

Classe	Ordem	Fam/Outros	Gên/Outros	Espécie	S-U	U-S	Zon.
Arachnida	Acari	Ixodidae	*Amblyomma*	sp.1		X	E
Arachnida	Araneae	Araneidae	*Alpaida*	sp.1	X		E
Arachnida	Araneae	Barychelidae		jovem		2	E
Arachnida	Araneae	Drymusidae		jovem		X	E
Arachnida	Araneae	Filistatidae		sp.1	X		E
Arachnida	Araneae	Ochyroceratidae		jovem		X	E
Arachnida	Araneae	Oonopidae	aff. *Xyccarph*	sp.2		X	E
Arachnida	Araneae	Pholcidae	*Leptopholcus*	sp.1		X	E
Arachnida	Araneae	Pholcidae	*Metagonia*	sp.	X		E
Arachnida	Araneae	Salticidae		jovem		X	E
Arachnida	Araneae	Scytodidae	Scytodes	sp.	X		E
Arachnida	Araneae	Segestriidae	*Ariadna*	sp.1		X	E
Arachnida	Araneae	Theridiidae	*Theridion*	sp.	X		E
Arachnida	Araneae	Theridiosomatidae	*Ogulnius*	sp.1		X	E
Arachnida	Araneae	Theridiosomatidae	*Plato*	sp.1	X	X	E
Arachnida	Opiliones	Cosmetidae		sp.	X		E
Arachnida	Pseudoscorpiones	Chernetidae	*Spelaeochernes*	sp.1	X	X	E
Arachnida	Pseudoscorpiones	Chthoniidae	*Pseudochthonius*	sp.1		X	E
Chilopoda	Geophilomorpha			sp.	X		E
Chilopoda	Scutigeromorpha	Pselliodidae	*Sphendononema*	*guildingii*	X		E
Chilopoda				jovem		1	E
Diplopoda	Polydesmida	Chelodesmidae		sp.	X		E
Entognatha	Collembola	Paronellidae		sp.1		X	E
Entognatha	Diplura	Projapygidae		sp.1		X	E
Insecta	Blattodea			jovem	X	1	E
Insecta	Coleoptera	Carabidae		sp.5		X	E
Insecta	Diptera	Aulacigastridae		sp.		X	E
Insecta	Diptera	Cecidomyiidae	Cecidomyiinae	sp.		X	E
Insecta	Diptera	Culicidae	*Culex*	sp.		X	E
Insecta	Diptera	Dolichopodidae		sp.		X	E
Insecta	Diptera	Psychodidae	Phlebotominae	sp.		X	E
Insecta	Diptera	Tipulidae	Tipulinae	sp.		X	E
Insecta	Heteroptera	Nabidae		sp.		X	E
Insecta	Heteroptera	Reduviidae	Emesinae	sp.	X	1	E
Insecta	Heteroptera	Reduviidae	*Zelurus*	sp.	X	1	E
Insecta	Homoptera	Cixiidae		sp.2		X	E
Insecta	Hymenoptera	Formicidae	*Camponotus*	sp.1		X	E
Insecta	Hymenoptera	Formicidae	*Dolichoderus*	aff. *bispinosus*	X	X	E
Insecta	Hymenoptera	Formicidae	*Gnamptogenys*	sp.1		X	E
Insecta	Hymenoptera	Formicidae	*Gnamptogenys*	*striatula*	X		E
Insecta	Hymenoptera	Formicidae	*Odontomachus*	*haematodus*	X		E
Insecta	Hymenoptera	Formicidae	*Pheidole*	sp.2		X	E
Insecta	Lepidoptera	Tineoidea		sp.3		X	E
Insecta	Neuroptera	Myrmeleontidae		jovem	X	X	E
Insecta	Orthoptera	Phalangopsidae	*Phalangopsis*	sp.	X		E
Insecta	Psocoptera			jovem		X	E
Gastropoda	Pulmonata	Subulinidae		sp.5		X	E
Gastropoda	Pulmonata	Systrophiidae	*Happia*	sp.		X	E
Malacostraca	Isopoda	Armadillidae		sp.1		X	E
Amphibia	Anura			sp.		1	E
Mammalia	Chiroptera	Emballonuridae	*Peropteryx*	sp.		1	E
Mammalia	Chiroptera			sp.	4		E
Mammalia	Rodentia	Cricetidae	*Rhipidomys*	sp.		1	E
